# Increase in CTGF mRNA expression by respiratory syncytial virus infection is abrogated by caffeine in lung epithelial cells

**DOI:** 10.1111/irv.12561

**Published:** 2018-05-22

**Authors:** Steffen Kunzmann, Christine Krempl, Silvia Seidenspinner, Kirsten Glaser, Christian P. Speer, Markus Fehrholz

**Affiliations:** ^1^ Clinic of Neonatology Buergerhospital Frankfurt am Main Frankfurt am Main Germany; ^2^ University Children's Hospital University of Wuerzburg Wuerzburg Germany; ^3^ Institute of Virology and Immunobiology University of Wuerzburg Wuerzburg Germany

**Keywords:** Caffeine, CCN2, dexamethasone, lung remodeling, poly(I:C)

## Abstract

Respiratory syncytial virus (RSV) is a leading cause of severe lower respiratory tract infection in early childhood. Underlying pathomechanisms of elevated pulmonary morbidity in later infancy are largely unknown. We found that RSV‐infected H441 cells showed increased mRNA expression of connective tissue growth factor (CTGF), a key factor in airway remodeling. Additional dexamethasone treatment led to further elevated mRNA levels, indicating additive effects. Caffeine treatment prevented RSV‐mediated increase in CTGF mRNA. RSV may be involved in airway remodeling processes by increasing CTGF mRNA expression. Caffeine might abrogate these negative effects and thereby help to restore lung homeostasis.

## 1 INTRODUCTION

Respiratory syncytial virus (RSV), a single‐strand negative sense RNA virus, is the most important cause of lower respiratory tract infection in early childhood^1^; especially in preterm neonates with chronic lung diseases like bronchopulmonary dysplasia (BPD), these infections can be clinically severe being associated with high morbidity and mortality. In addition, RSV increases the long‐term risk for subsequent wheezing and elevated susceptibility to asthma. Underlying molecular mechanisms are not yet fully known.^2^

Connective tissue growth factor (CTGF), a downstream mediator of transforming growth factor (TGF)‐β, is a matricellular signaling modulator and a key molecule in tissue remodeling.^3^ As upregulation of CTGF has been detected after mechanical ventilation and hyperoxia in the neonatal lung,^4^ in proliferating type II lung epithelial cells, and activated fibroblasts of fibrotic lungs,^5^ it has been suggested that CTGF may contribute to BPD. Moreover, a role of RSV in activation of TGF‐β gene expression has been described,^6^ suggesting a possible involvement of CTGF during RSV infection.

Administration of glucocorticoids to attenuate severe forms of BPD in preterm infants is controversially discussed,^7^ and the potential impacts of glucocorticoids as well as RSV infection on remodeling processes of the lung are barley defined. Caffeine, a methylxanthine commonly used to minimize apnea of prematurity, has been associated with the ability to reduce the incidence of BPD.^8^

We recently described increase in CTGF expression by various glucocorticoids in lung epithelial and fibroblast models.^9^ In addition, we have shown that TGF‐β1‐induced upregulation of CTGF can be antagonized by caffeine in A549 lung epithelial cells.^10^ As anti‐inflammatory agents appear to offer no clinical benefit,^11^ the interplay of inflammatory trigger, inflammation, and novel agents positively influencing remodeling processes of the lung is the subject of intense research.

We hypothesized that caffeine might counteract detrimental effects of glucocorticoids and RSV infection. Using the human lung epithelial cell line H441 and the fetal lung fibroblast strain IMR‐90, the aim of this study was to define if RSV has an impact on the expression of CTGF mRNA in the simultaneous presence of dexamethasone and caffeine.

## 2 MATERIAL AND METHODS

### 2.1 Reagents

Caffeine and dexamethasone were purchased from Sigma‐Aldrich (St. Louis, CA). Polyinosinic:polycytidylic acid (poly(I:C)) was from InvivoGen (San Diego, CA). Linear polyethylenimine MW 25 000 (PEI) was purchased from Polysciences Inc. (Warrington, PA).

### 2.2 Cells and virus

NCI‐H441 (H441) and IMR‐90 cells were purchased from ATCC (LGC Standards, Teddington, UK) and cultured as described.^9^ Recombinant green fluorescent protein‐expressing RSV (rgRSV) was a kind gift of Peter L. Collins (National Institute of Allergy, Immunology, and Infectious Diseases, National Institutes of Health, Bethesda, MD).

### 2.3 Infection and transfection

For infection, cells were seeded on six‐well plates (Greiner, Frickenhausen, Germany) until 80% confluence was reached and infected with 5 × 10^5^ pfu of rgRSV. After 2 hour, medium was removed and cells were incubated with substances in growth medium as indicated. Images of cells were captured using a DM IRE 220 microscope (Leica, Solms, Germany). For transfection, 5 × 10^5^ cells were seeded on six‐well plates (Greiner) and 16 hour later transfected with 10 μg poly(I:C) using 20 μg PEI in a total volume of 1 mL Opti‐MEM I (Gibco, Thermo Fisher Scientific, Waltham, MA). After 4 hour, medium was removed and cells were treated for 24 hour with substances in growth medium as indicated.

### 2.4 RNA extraction, RT‐PCR, and quantitative real‐time PCR (qPCR)

RNA extraction, RT‐PCR, and qPCR were performed as described previously.^9^ Levels of mRNAs were normalized to those of glyceraldehyde‐3‐phosphate dehydrogenase (GAPDH). Primer sequences for qPCR are listed in Table 1. Mean fold changes in mRNA expression were calculated by the ΔΔC_T_ method.

**Table 1:** Primers for qPCR

Gene symbol	Sequence accession #	Orientation	Sequence [5′ to 3′]	Amplicon length [bp]
CTGF	NM_001901.2	Forward	ACCCAACTATGATTAGAGCC	189
		Reverse	TTGCCCTTCTTAATGTTCTC	
GAPDH	NM_002046.5	Forward	CCATGGAGAAGGCTGGGG	195
		Reverse	CAAAGTTGTCATGGATGACC	
IL6	NM_000600.4	Forward	AACAAATTCGGTACATCCTC	167
		Reverse	AAGTCTCCTCATTGAATCCA	
CXCL8 (IL8)	NM_000584.3	Forward	CAGTGCATAAAGACATACTCC	198
		Reverse	TTTATGAATTCTCAGCCCTC	

CTGF, Connective tissue growth factor.

### 2.5 Statistical analysis

Results are given as means ± SD. Unless otherwise stated, data were analyzed using one way ANOVA with Bonferroni's multiple comparison post hoc test. A *P*‐value ≤.05 was considered significant. All statistical analyses were performed using Prism^®^ version 6 (GraphPad Software, San Diego, CA).

## 3 RESULTS

### 3.1 Impact of rgRSV and dexamethasone on inflammatory markers in H441 and IMR‐90 cells

**Figure 1:**
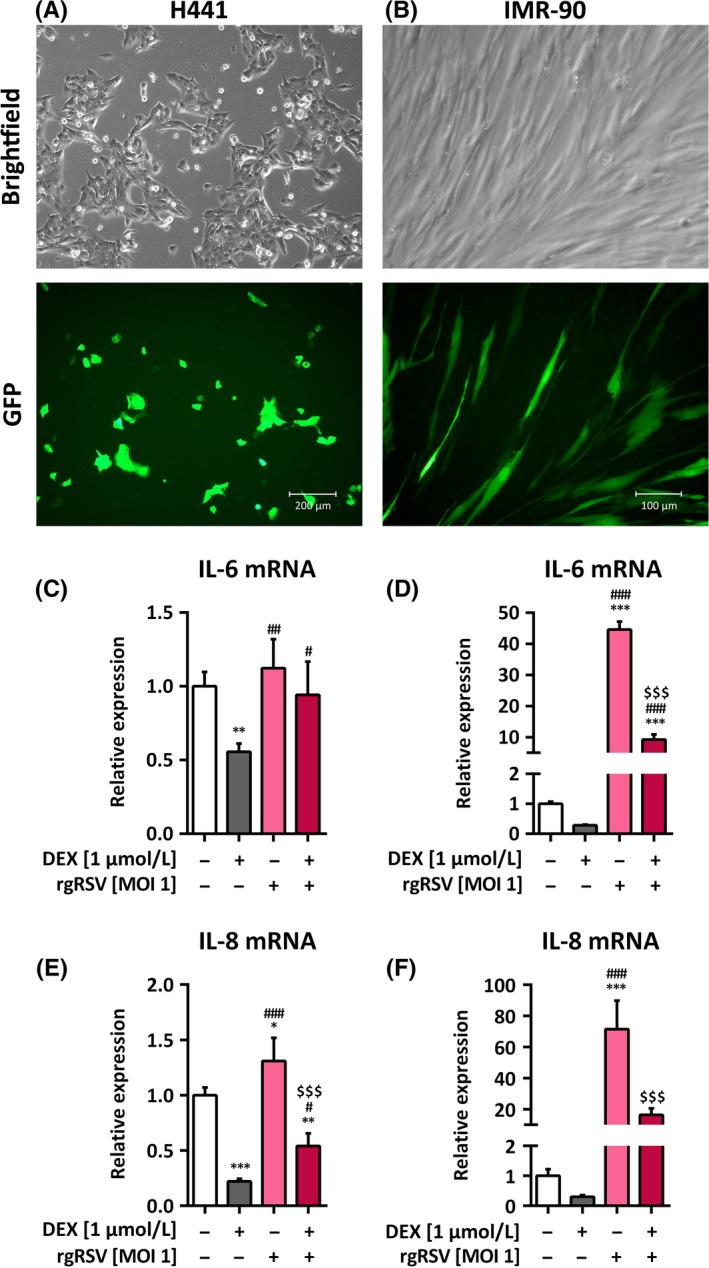
Infection of H441 and IMR‐90 cells with rgRSV and increase in inflammatory markers. H441 and IMR‐90 were either left untreated or infected with rgRSV and after 2 hour treated with 1 μmol/L dexamethasone for 24 hour. qPCR specific for IL‐6 and IL‐8 mRNA was performed. Images of H441 (A) and IMR‐90 cells (B) 24 hour post‐infection with rgRSV. Virus infection is visible by expression of GFP (lower panel), cell morphology of infected cells in brightfield (upper panel). IL‐6 (C and D) and IL‐8 (E and F) mRNA levels of H441 (C and E) and IMR‐90 cells (D and F), respectively, were normalized to GAPDH mRNA and fold differences compared to corresponding untreated cells were calculated. Means + SD of n = 4 independent experiments are shown. *P <* *.05; **P <* *.01; ***P <* *.001 compared to untreated controls; ^#^ *P *<* *.05; ^##^ *P *<* *.01; ^###^ *P *<* *.001 compared to cells treated with dexamethasone; ^$$$^ *P *<* *.001 compared to cells infected with rgRSV

After treatment of lung epithelial cells H441 and fetal lung fibroblasts IMR‐90 with rgRSV for 24 hour, virus infection was evident to varying degrees in all lung cell cultures by the expression of GFP (Figure 1A and B). While no increase in IL‐6 mRNA expression could be observed in H441 cells (Figure 1C), rgRSV infection increased IL‐6 mRNA expression in IMR‐90 cells (44.6 ± 2.5‐fold, *P *<* *.0001; Figure 1D). IL‐8 mRNA expression was increased in H441 (1.3 ± 0.2‐fold, *P *=* *.0243; Figure 1E) as well as in IMR‐90 cells (71.5 ± 18.4‐fold, *P *<* *.0001; Figure 1F). Dexamethasone was able to significantly reduce this rgRSV‐mediated increase in IL‐6 mRNA in IMR‐90 cells (*P *<* *.0001) and of IL‐8 mRNA in H441 and IMR‐90 cells (*P *<* *.0001 for both cell types).

### 3.2 Effect of rgRSV, dexamethasone, and caffeine on expression of CTGF mRNA in H441 and IMR‐90 cells

**Figure 2:**
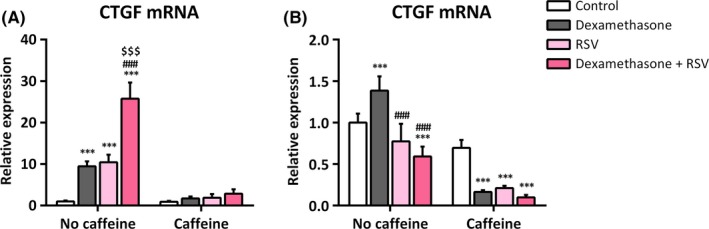
Increase in connective tissue growth factor (CTGF) mRNA expression by dexamethasone and rgRSV in H441 and by dexamethasone in IMR‐90 cells is abrogated by caffeine treatment. H441 and IMR‐90 cells were either left untreated or infected with rgRSV and after 2 hour treated with 1 μmol/L dexamethasone and/or 10 mmol/L caffeine for 24 hour. qPCR of CTGF mRNA was performed, CTGF mRNA levels of H441 cells (A) and IMR‐90 cells (B) were normalized to GAPDH mRNA, and fold differences compared to untreated cells were calculated. Means + SD of n ≥ 3 independent experiments are shown. **P <* *.01; ***P <* *.001 compared to corresponding controls; ^###^ *P *<* *.001 compared to corresponding cells treated with dexamethasone; ^$$$^ *P *<* *.001 compared to corresponding cells infected with rgRSV

Infection with rgRSV was able to increase CTGF mRNA expression in H441 (10.5 ± 1.8‐fold, *P *=* *.0002; Figure 2A) but not in IMR‐90 cells (Figure 2B). Dexamethasone increased CTGF mRNA expression in H441 (9.5 ± 1.2‐fold, *P *=* *.0007; Figure 2A) and in IMR‐90 cells (1.4 ± 0.2‐fold, *P *=* *.0006; Figure 2B). In combination, dexamethasone and rgRSV showed an additive increase in H441 cells (25.7 ± 3.9‐fold, *P *<* *.0001; Figure 2A) but reduced CTGF mRNA levels in IMR‐90 cells (0.6 ± 0.1‐fold, *P *=* *.0003; Figure 2B). In H441 cells, the presence of caffeine was able to prevent a significant increase in CTGF mRNA by dexamethasone, rgRSV, and their combination (Figure 2A). Dexamethasone‐induced increase in CTGF mRNA in IMR‐90 cells was prevented in the presence of caffeine. In addition, CTGF mRNA levels in comparison with cells treated with caffeine alone were further reduced by dexamethasone, rgRSV, and their combination (*P *<* *.0001 for all comparisons; Figure 2B).

### 3.3 Poly(I:C) increases mRNA expression of inflammatory markers and CTGF in H441 cells

**Figure 3:**
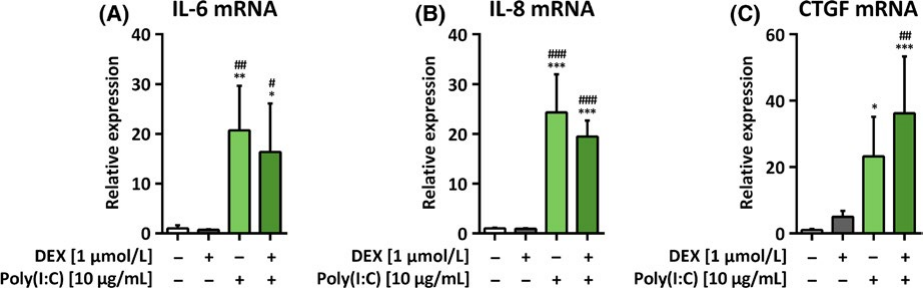
Poly(I:C) increases mRNA expression of inflammatory markers and connective tissue growth factor (CTGF) in H441 cells. H441 cells were either treated with PEI alone or transfected with 10 μg/mL poly(I:C) and after 4 hour treated with 1 μmol/L dexamethasone for 24 hour. qPCR against IL‐6 (A) IL‐8 (B), and CTGF (C) mRNA was performed. mRNA levels were normalized to GAPDH and fold differences compared to untreated cells were calculated. Means + SD of n = 3 independent experiments are shown. *P <* *.05; **P <* *.01; ***P <* *.001 compared to untreated controls; ^#^ *P *<* *.05; ^##^ *P *<* *.01; ^###^ *P *<* *.001 compared to cells treated with dexamethasone

24 hour after transfection of H441 cells with poly(I:C), IL‐6 (20.7 ± 8.9‐fold, *P *=* *.0014; Figure 3A), IL‐8 (24.3 ± 7.7‐fold, *P *<* *.0001; Figure 3B), and CTGF mRNA expression was increased (23.2 ± 12.0‐fold, *P *=* *.0245; Figure 3C). Although CTGF mRNA levels were further elevated by the addition of dexamethasone in poly(I:C)‐transfected cells (36.2 ± 17.1‐fold), this was not statistically significant (*P *=* *.3992) in comparison with cells transfected with poly(I:C) alone.

## 4 DISCUSSION

We report an upregulation of CTGF mRNA by RSV infection in H441 lung epithelial cells which was additive to the recently described induction by glucocorticoids.^9^ Different to work describing a reduction of vanadium pentoxide‐induced fibrosis by RSV via reduction of growth factors,^12^ our data indicate that RSV infection might conceivably be able to induce fibrotic conditions by increasing CTGF mRNA in lung epithelial cells which might be further enhanced by glucocorticoids.^3^, ^13^

It has been shown that RSV‐induced exacerbation of airway hyperresponsiveness in a mouse model was only partially suppressed by steroid treatment.^14^ According to our observations in H441 cells, the simultaneous presence of glucocorticoids and RSV infection might provoke aggravated remodeling processes in the lower airways via increase in CTGF expression, which has been demonstrated in bleomycin‐induced fibrosis.^13^ Furthermore, in addition to the prevention of glucocorticoid‐mediated increase,^9^ our data suggest that caffeine treatment is able to block RSV—as well as dexamethasone/RSV‐caused upregulation of CTGF mRNA expression. Apart from its preventive effects on the development of BPD,^8^ caffeine might thus also counteract potential pro‐fibrotic effects mediated by RSV.

Poly(I:C), an artificial dsRNA structure, stimulates toll‐like receptor‐3 signaling and induces an activation of the RNA helicases RIG‐I and MDA‐5, thereby initiating cytokine production.^15^ By transfecting H441 cells with poly(I:C), mimicking viral dsRNA replication intermediates, we reveal that the observed increase in CTGF mRNA can be mediated by cytoplasmic dsRNA structures. In contrast to infection with rgRSV, IL‐6 and IL‐8 mRNA levels were much stronger elevated in the presence of poly(I:C), possibly indicating anti‐inflammatory mechanisms mediated by RSV predominantly in H441 cells, as the inflammatory outcome in IMR‐90 cells was more pronounced.

However, there are certain limitations of this study to be considered. The fact that increase in CTGF mRNA expression by rgRSV infection in contrast to that by dexamethasone treatment could be observed exclusively in H441 cells indicates a possible restriction to lung epithelial cells, a feature of papillary adenocarcinoma cells, or a consequence of cytopathogenicity. In addition, the time at which a maximum of infection could be reached was too early to detect CTGF protein expression in H441 cells.

## 5 CONCLUSION

We report a transcriptional attribute of increased CTGF mRNA expression in H441 cells following infection with RSV potentially prevalent for and restricted to lung epithelial cells. Caffeine might have the ability to support anti‐inflammatory actions of glucocorticoids but antagonize initiation of CTGF expression, thereby pushing the overall outcome from long‐term morbidity to healing.

## References

[1] Openshaw PJM , Chiu C , Culley FJ , Johansson C . Protective and harmful immunity to RSV infection. Annu Rev Immunol. 2017;35:501‐532. 28226227 10.1146/annurev-immunol-051116-052206

[2] Piedimonte G . RSV infections: state of the art. Cleve Clin J Med. 2015;82:03. 10.3949/ccjm.82.s1.03 26555808

[3] Kubota S , Takigawa M . Cellular and molecular actions of CCN2/CTGF and its role under physiological and pathological conditions. Clin Sci. 2015;128:181‐196. 25294165 10.1042/CS20140264

[4] Alapati D , Rong M , Chen S , et al. Connective tissue growth factor antibody therapy attenuates hyperoxia‐induced lung injury in neonatal rats. Am J Respir Cell Mol Biol. 2011;45:1169‐1177. 21659659 10.1165/rcmb.2011-0023OC

[5] Pan LH , Yamauchi K , Uzuki M , et al. Type II alveolar epithelial cells and interstitial fibroblasts express connective tissue growth factor in IPF. Eur Respir J. 2001;17:1220‐1227. 11491168 10.1183/09031936.01.00074101

[6] Mgbemena V , Segovia J , Chang T , Bose S . Kruppel‐like factor 6 regulates transforming growth factor‐beta gene expression during human respiratory syncytial virus infection. Virol J. 2011;8:8‐409. 21849067 10.1186/1743-422X-8-409 PMC3170303

[7] Rostas SE , McPherson C . Systemic corticosteroids for the prevention of Bronchopulmonary Dysplasia: picking the right drug for the right baby. Neonatal Netw. 2016;35:234‐239. 27461202 10.1891/0730-0832.35.4.234

[8] Abdel‐Hady H , Nasef N , Shabaan AE , Nour I . Caffeine therapy in preterm infants. World J Clin Pediatr. 2015;4:81‐93. 26566480 10.5409/wjcp.v4.i4.81 PMC4637812

[9] Fehrholz M , Glaser K , Speer CP , Seidenspinner S , Ottensmeier B , Kunzmann S . Caffeine modulates glucocorticoid‐induced expression of CTGF in lung epithelial cells and fibroblasts. Respir Res. 2017;18:017‐0535. 10.1186/s12931-017-0535-8 PMC5363056 28330503

[10] Fehrholz M , Speer CP , Kunzmann S . Caffeine and rolipram affect smad signalling and TGF‐β1 stimulated CTGF and transgelin expression in lung epithelial cells. PLoS One. 2014;9:e97357. 24828686 10.1371/journal.pone.0097357 PMC4020861

[11] Chambers RC , Mercer PF . Mechanisms of alveolar epithelial injury, repair, and fibrosis. Ann Am Thorac Soc 2015;12:S16‐S20. 25830828 10.1513/AnnalsATS.201410-448MG PMC4430974

[12] Turpin EA , Antao‐Menezes A , Cesta MF , et al. Respiratory syncytial virus infection reduces lung inflammation and fibrosis in mice exposed to vanadium pentoxide. Respir Res. 2010;11:20. 20175905 10.1186/1465-9921-11-20 PMC2841591

[13] Yang J , Velikoff M , Canalis E , Horowitz JC , Kim KK . Activated alveolar epithelial cells initiate fibrosis through autocrine and paracrine secretion of connective tissue growth factor. Am J Physiol Lung Cell Mol Physiol. 2014;306:7. 10.1152/ajplung.00243.2013 PMC3989723 24508728

[14] Nguyen TH , Maltby S , Simpson JL , et al. TNF‐alpha and macrophages are critical for respiratory syncytial virus‐induced exacerbations in a mouse model of allergic airways disease. J Immunol. 2016;196:3547‐3558. 27036916 10.4049/jimmunol.1502339

[15] Hafner AM , Corthesy B , Merkle HP . Particulate formulations for the delivery of poly(I:C) as vaccine adjuvant. Adv Drug Deliv Rev. 2013;65:1386‐1399. 23751781 10.1016/j.addr.2013.05.013

